# Closing the implementation gap: a harmonised EU framework for synthetic nucleic acid screening and customer verification

**DOI:** 10.3389/fbioe.2026.1819942

**Published:** 2026-07-01

**Authors:** Sophie Peresson, Patrick Stadler

**Affiliations:** 1 Independent Consultant, Paris, France; 2 Pour Demain, Bern, Switzerland

**Keywords:** biosecurity, know your customer (KYC) verification, regulation, sequence screening, sequences of concern (SOC)

## Abstract

Synthetic nucleic acid technologies underpin modern biological research and biomanufacturing. However, their growing accessibility through commercial synthesis services, benchtop devices, and AI-assisted design tools creates significant dual-use biosecurity risks. This paper presents a harmonised EU biosecurity screening framework operationalised through the proposed EU Biotech Act I (December 2025), evaluating two complementary control mechanisms: Know Your Customer (KYC) verification and sequence screening against Sequences of Concern (SOC). We find that existing voluntary frameworks leave systematic coverage gaps that binding EU-level obligations, anchored to ISO 20688-2:2024, would substantively address — particularly for smaller providers and benchtop devices. We argue that near-term EU action offers a time-sensitive opportunity to shape global synthetic biology governance before regulatory fragmentation becomes entrenched.

## 1 Introduction

Synthetic nucleic acid technologies—DNA and RNA synthesis underpin European research, health security, and biomanufacturing, enabling rapid vaccine development, sustainable bioproduction, and major advances in diagnostics and therapeutics. These technologies sit at the core of the EU Biotech Act’s general objective (European Commission, 2025) to strengthen the Union’s biotechnology research, development and production capabilities, reinforce competitiveness, support manufacturing resilience, facilitate AI integration, and prevent misuse of biotechnology while safeguarding the internal market. Several initiatives are working to promote a secure innovation ecosystem for providers of nucleic acid services. The International Biosecurity and Biosafety Initiative for Science (IBBIS) provides free, distributed, open-source automated software for screening sequences of nucleic acids as well as customer screening resources through its Common Mechanism project, and leads the DNA Screening Standards Consortium (DSSC) to drive global harmonisation of screening standards (IBBIS, 2024;IBBIS, 2025; Wheeler et al., 2024). The Engineering Biology Research Consortium (EBRC), in partnership with NIST, has conducted extensive stakeholder engagement to develop robust tools, capabilities, and standards for nucleic acid synthesis screening, and published a comprehensive report on strengthening a safe and secure nucleic acid synthesis ecosystem (EBRC, 2025). These initiatives represent important complementary efforts to the regulatory frameworks discussed in this paper.

However, their availability through commercial synthesis services, benchtop devices, and AI-assisted design tools raises serious dual-use biosecurity concerns.

These risks are not hypothetical: documented cases include the reconstruction of the 1918 influenza virus from synthetic DNA, the synthesis of horsepox virus, and demonstrations by journalists that select agent sequences could be ordered without verification. As detailed in the RAND Corporation’s analysis of securing commercial nucleic acid synthesis (Crawford et al., 2024, pp. 19–20), commercial gene synthesis presents a serious risk of deliberate misuse or accident, with AI tools compounding this risk by potentially enabling the design of novel proteins that evade existing screening methods. At the same time, the transformative nature of these innovations serves to advance bioeconomy and equity goals globally — from enabling low-cost vaccine production in resource-limited settings to supporting sustainable biomanufacturing. Embedding responsible nucleic acid synthesis screening (NASS) and biosecurity practices as a key enabler, rather than a constraint, is essential for the responsible harnessing of these technologies. If unaddressed, the EU faces regulatory fragmentation, competitive disadvantages, and avoidable security vulnerabilities — risks that fall within the concept of ‘biotechnology products of concern’ under the proposed European Biotech Act.

We present these controls as practical mechanisms to operationalize Chapter VIII (Biodefence and Preventing Biotechnology Misuse) of the EU Biotech Act I – formally proposed on 16 December 2025 – while maintaining proportionality and safeguarding innovation, translating its provisions into implementable controls through implementing and delegated acts adopted in accordance with Articles 25a and 64 of the Regulation. Chapter VIII provides the legal basis for screening, verification of legitimate need, and the reporting and tracking of suspicious transactions involving biotechnology products of concern listed in Annex I, supported by enforcement mechanisms to ensure compliance.

Finally, the proposed controls are consistent with relevant international standards and initiatives, including ISO 20688-2:2024, the IGSC (IGSC, 2024) protocol, and guidance from the WHO and OECD. They also complement recent global efforts such as the Biosecurity Modernization and Innovation Act (Cotton and Klobuchar, 2026) and the International Bio Funders Compact launched by CEPI and NTI (CEPI, 2024) and Executive Order 14292 on improving the safety and security of biological research (The White House, 2025).

## 2 Recommendations and rationale

Building on Chapter VIII of the EU Biotech Act I and the implementation challenges identified above, this section sets out a coherent package of legally grounded measures, proportionate to risk, and compatible with innovation and internal market objectives.

### 2.1 Establish EU-Wide screening obligations

Chapter VIII of the EU Biotech Act I provides the legal foundation for harmonised KYC and sequence screening obligations across the EU. These obligations should be embedded through delegated and implementing acts (as well as guidelines), anchored in the Act’s provisions on verification of legitimate need and suspicious transaction reporting. Coordination with Regulation (EU) 2021/821 (European Parliament and Council of the European Union, 2021) on dual-use export controls is essential to prevent duplication where biotechnology products are already subject to existing controls. Harmonised EU-wide obligations would substantially outperform fragmented national measures. They would strengthen legal certainty, reduce compliance duplication, and protect internal market integrity. A single EU baseline would enable economies of scale in screening tools and SOC management while reducing uncertainty for SMEs and benchtop manufacturers. Divergent national thresholds impose compliance costs that a common framework would eliminate. Importantly, current standards were developed primarily for DNA synthesis and do not fully address RNA synthesis — a gap acknowledged in the literature given that the 2023 HHS Screening Framework Guidance expanded recommendations to cover RNA (U.S. Department of Health and Human Services, Administration for Strategic Preparedness and Response, 2023), but ISO 20688-2:2024 itself specifies requirements for synthesised gene fragments, genes, and genomes in the form of double-stranded DNA (International Organization for Standardization, 2024). As noted in the RAND report on securing commercial nucleic acid synthesis (Crawford et al., 2024), screening protocols vary across providers and existing frameworks have not comprehensively addressed the RNA synthesis landscape. Implementing acts should explicitly confirm whether existing screening methodologies are adequate for RNA and establish interim measures where gaps remain.

Implementing acts should also clarify how obligations apply to non-EU providers supplying synthesis services into the EU market, to prevent regulatory arbitrage.

Industry-led initiatives—most notably the International Gene Synthesis Consortium (IGSC)—have played a crucial leadership role. However, voluntary schemes alone cannot ensure universal coverage. A binding EU baseline would close coverage gaps and reduce intra-EU forum shopping.

Experience with U.S. screening guidance shows voluntary uptake improves awareness and best practices but leaves uneven implementation across smaller or non-aligned providers (NSABB, 2023). Harmonised legal obligations more effectively ensure comprehensive baseline safeguards, particularly as distributed manufacturing and new entrants expand the market.

EU codification aligned with ISO 20688-2:2024 and emerging frameworks (OSTP, 2024; UK Government, 2024; OECD, 2025) would enhance predictability and strengthen the EU’s international norm-setting role, generating positive regulatory convergence effects while reducing long-term compliance uncertainty.

Delegated acts preserve proportionality by allowing technical requirements—such as SOC list updates informed by the Advisory Group on Biosecurity’s monitoring of biological risks—to evolve without reopening primary legislation, maintaining regulatory agility while protecting internal market integrity.

### 2.2 Align with standards and ensure interoperability

EU obligations should interoperate with, rather than duplicate, the international standard-setting ecosystem already developed around biosecurity screening. ISO 20688-2:2024 (which covers sequence screening for nucleic acid synthesis) and the IGSC Harmonized Protocol (IGSC, 2024) are the most widely adopted frameworks for sequence screening and customer verification, and many providers are already investing in compliance with them. A wholly separate domestic standard would impose redundant compliance costs and fragment the global screening landscape.

Anchoring EU obligations to these established frameworks — while preserving Commission authority to set minimum outcome requirements — would reduce friction for cross-border providers, create conditions for mutual recognition with like-minded jurisdictions, and position the EU as a constructive norm-setter. It would also future-proof the framework: as ISO and IGSC standards evolve, EU obligations aligned to them can adapt without primary legislative revision.

### 2.3 Integrating SOC lists into layered biosecurity governance

The EU should establish a centrally managed sequences-of-concern (SOC) service under Commission oversight. This service would provide a curated list, version control, and secure API access with tiered sensitivity levels and robust access controls. Updates should be informed by the Advisory Group on Biosecurity and may be reflected, where appropriate, through delegated acts updating Annex I.

To mitigate risks of impeding legitimate research, the development of implementing acts and SOC list curation should include structured consultation mechanisms engaging the broader academic and life sciences research community beyond the Advisory Group on Biosecurity.

Without a single authoritative source, providers, national authorities, and the Commission would each risk operating against different or outdated sequence lists — undermining the consistency that layered governance depends on.

From a privacy and cybersecurity perspective, robust technical safeguards protect sensitive sequence data while ensuring regulatory compliance with existing EU frameworks. On the international stage, demonstrating responsible stewardship of dual-use biological information enhances the EU’s credibility as a standard-setter in global biosecurity governance, facilitating partnerships and information exchange with third countries and international organizations.

The SOC service should also support projects contributing to the EU Biothreat Radar by enabling pathogen-agnostic detection, cross-border surveillance interoperability, and data sharing consistent with internationally recognised pathogen data standards — capabilities that are critical to strengthening EU preparedness for future pandemic, epidemic, and outbreak response, as well as medical countermeasure (MCM) development.

### 2.4 Implement risk-based escalation and reporting

The EU should require a standardized decision tree for SOC matches offering graduated responses (accept/deny/request additional information/refer to authorities) that aligns with Chapter VIII’s risk-based approach to biosecurity governance. This framework should incorporate the reporting of suspicious transactions to designated national contact points and include safe harbor protections to encourage compliance without stifling legitimate research activities (Table 1).

**TABLE 1:** Mapping EU legal instruments to screening functions.

Screening function	Primary instrument	Role in implementation	Notes	Date/Year of instrument
Baseline obligations	EU Biotech Act	Establish EU-wide minimum KYC: sequence screening	Delegated acts for updates	Proposed 16 December 2025
Export control interface	Reg. 2021/821	Align SOC treatment with dual use controls	Clarify definitions/scope	Adopted 20 May 2021; in force 9 September 2021
Data protection	GDPR (Reg. 2016/679)	Data minimisation, DPIA, retention	Joint guidance with EDPB	Adopted 27 April 2016; applicable from 25 May 2018
Cybersecurity	NIS2 (Dir. 2022/2555)	Security by design for screening systems	Logging incident reporting	Adopted 14 December 2022; transposition deadline 17 October 2024
Device security	Product safety/cyber rules	Embedded screening/user authentication	Phased compliance	Ongoing; EU Cyber Resilience Act adopted October 2024
AI oversight and biorisk	EU AI Act (Reg. 2024/1689)	Oversight of AI models with biological systemic risk; assessment of AI capabilities in biodesign	Advisory Group interface; high-risk AI system classification relevant to synthesis screening tools	Adopted 13 June 2024; phased application from 2 August 2024

Clear protocols would enhance operational predictability by reducing provider liability concerns and inconsistent practices, encouraging systematic risk assessment rather than precautionary over-blocking. A graduated escalation mechanism ensures proportionate responses by enabling context-appropriate interventions instead of blanket denials, thereby preserving legitimate research activities while addressing genuine security concerns. From a continuous improvement perspective, aggregated, privacy-preserving analytics can feed into SOC curation refinement and guidance development, creating a learning system that adapts to evolving threat landscapes while maintaining transparency and accountability.

### 2.5 Design hybrid conformity assessment: certification, audit, and self-attestation

The EU should introduce a tiered conformity assessment model combining self-attestation, third-party certification, and audits, aligned with the Biotech Act’s emphasis on proportionality, SME accessibility, and internal market coherence. Audit frequency and rigor should be tailored to risk profiles and organizational maturity. An EU registry of compliant providers could enhance market transparency and build customer confidence.

The conformity assessment model should be designed as a functionally independent mechanism from the Advisory Group on Biosecurity, while drawing on its technical outputs. The Advisory Group’s mandate is scientific threat assessment and monitoring — advising on emerging biological risks and AI capabilities posing biosystemic concerns. Conformity assessment, by contrast, is an operational and market-facing function concerned with verifying that providers and manufacturers meet defined minimum standards. Conflating these roles risks undermining both the scientific integrity of threat assessment and the operational integrity of compliance verification. The conformity assessment body should therefore operate under Commission oversight, with defined referral and information-sharing protocols to the Advisory Group, rather than as a sub-function of it. This separation is consistent with the Act’s general architecture, which distinguishes advisory (Chapter X) from implementing (Chapter VIII) functions. Drawing on analogous arrangements under the AI Act (European Parliament and Council of the European Union, 2024) and the Medical Devices Regulation, where notified bodies conduct conformity assessment independently of scientific committees, the EU should designate accredited national bodies or a dedicated EU-level entity for this function.

A tiered approach would improve scalability by enabling efficient regulatory oversight across diverse provider types—from multinational corporations to academic facilities—without overwhelming enforcement capacity. Certification becomes a competitive differentiator, creating positive incentives for compliance while allowing customers to make informed choices about their suppliers. For SMEs and research institutions, risk-based tailoring ensures accessibility by avoiding disproportionate administrative burdens, supporting both innovation and security objectives.

### 2.6 Cover benchtop devices and in-house synthesis

The EU should require embedded screening capabilities and user verification in benchtop synthesizers where such devices fall within or enable access to biotechnology products of concern listed in Annex I to the Biotech Act, specifically benchtop nucleic acid synthesis equipment capable of creating sequences of concern.

In line with the Act’s recommendations (Chapter IX and Articles 49a–49b), Member States or the Commission may establish regulatory sandboxes to test embedded screening technologies, alternative compliance mechanisms, or supervisory approaches prior to full-scale implementation, particularly where harmonised legal frameworks are still evolving.

For new benchtop devices, manufacturers should integrate screening capabilities at the design stage, with full compliance required prior to market entry. For existing or older benchtop devices (retrofits), implementation should account for technical and operational constraints, including external screening modules, firmware updates where architecturally feasible, or institutional-level screening protocols that intercept synthesis requests before device execution. Extended compliance timelines for retrofits should reflect the technical challenges of adapting legacy systems, with potential exemptions for devices nearing end-of-life or those with prohibitive modification costs, subject to enhanced institutional oversight requirements.

There is an emerging and important role for metadata screening in the governance of benchtop devices. As Rose et al. (2024) note in their analysis of practical questions for securing nucleic acid synthesis, benchtop synthesizers fundamentally bypass centralised screening by enabling customers to ‘print’ DNA in their own labs, vastly increasing the potential for misuse. Metadata screening — the analysis of non-sequence data associated with synthesis requests such as order patterns, user credentials, device identifiers, institutional affiliations, and temporal patterns—provides a complementary layer of oversight that does not require sequence-level analysis at the point of synthesis. A 2024 study on securing benchtop DNA synthesizers (Institute for Progress, 2024) highlights that devices could report metadata to secure cloud-based tools or screening software embedded in firmware to flag anomalous behaviour. Such metadata approaches are particularly valuable for detecting split-order strategies — where sequences of concern are distributed across multiple orders or devices to avoid triggering sequence-level alerts — and for monitoring devices that may have been tampered with or that operate in low-connectivity environments. Implementing acts should therefore require manufacturers to design benchtop devices capable of logging and reporting relevant metadata, subject to GDPR (European Parliament and Council of the European Union, 2016)-compliant data minimisation and retention requirements.

Phased compliance timelines, technical assistance programs for original equipment manufacturers, and standardized institutional standard operating procedure templates should support practical adoption across both new and retrofit scenarios. Embedding screening capabilities in benchtop devices addresses decentralization and portability risks as synthesis technology becomes more accessible.

### 2.7 Provide economic support and phased implementation

The EU should provide support through e.g., grants, procurement incentives, particularly for SMEs and public laboratories to prevent competitive distortions arising from new biosecurity obligations. Phased compliance timelines should be matched to risk profiles and organizational capacity. Eligibility preferences in EU research programs—such as the next Horizon Europe framework programme and the European Competitiveness Fund—could be extended to compliant actors. Synergies with Regulation (EU) 2024/795 (STEP), which treats health biotechnology strategic projects as contributing to STEP objectives for strategic technologies support, should be leveraged to maximize impact.

Financial and technical support would promote equity and uptake by reducing barriers for resource-constrained entities, accelerating sector-wide convergence toward biosecurity best practices. Such measures preserve innovation incentives by rewarding responsible actors rather than penalizing early adopters, mitigating cost concerns during transition periods and preventing market distortions. Targeted support also enables capacity building through knowledge transfer and capability development, particularly in Member States with emerging biotechnology sectors, strengthening the EU’s overall biosecurity posture while maintaining a level playing field across the internal market.

### 2.8 Clarify data protection, cybersecurity, and liability

The European Commission, European Data Protection Board, and ENISA could consider publishing a joint guidance covering GDPR-compliant KYC implementation, data minimization principles, retention limits, and security-by-design requirements for screening systems. The guidance could clarify that screening service providers and SOC database operators fall within the scope of Directive NIS2 (European Parliament and Council of the European Union, 2022) as essential or important entities and must comply with cybersecurity risk-management measures (Article 21), incident reporting obligations (Article 23), and supervision/enforcement requirements (Articles 32–33). Safe harbor protections for good-faith screening and reporting should be established, and coordination with export control obligations under Regulation (EU) 2021/821 (European Parliament and Council of the European Union, 2021) should be specified to avoid duplication where biotechnology products are subject to dual-use controls.

Clear guidance would enhance legal certainty by defining privacy obligations and liability protections, encouraging systematic reporting and cross-border collaboration while reducing regulatory hesitancy among economic operators. Transparent data stewardship strengthens societal confidence in biosecurity measures, which is essential for maintaining the social license for synthetic biology innovation. Explicit treatment of overlaps between data protection, cybersecurity, export control, and biosecurity regimes prevents implementation conflicts and compliance gaps that could undermine both security objectives and fundamental rights protections.

## 3 Concrete actions

The preceding sections established the legal and governance rationale for embedding KYC and sequence screening within Chapter VIII of the EU Biotech Act. This section translates those arguments into a sequenced set of near-term actions grounded in three principles: legal clarity before technical infrastructure; proportionate, capacity-sensitive implementation; and legitimacy through transparency, review, and oversight.

The RAND Europe cost-benefit analysis of synthetic nucleic acid screening in the EU (Zakaria et al., 2026, Table 3, p. 36) provides important evidence on the economic case for mandatory screening: the analysis models three policy approaches over a 10-year horizon and finds that mandatory EU-wide regulation delivers the largest societal benefit (€4.6 billion annually), generating roughly €6 in avoided losses per €1 spent, while voluntary measures deliver only modest gains (€193 million annually). These findings strongly support the case for binding obligations over voluntary approaches and underscore the importance of proportionate but comprehensive implementation.

### 3.1 Establish the legal baseline (immediate)

The first priority is the adoption of implementing acts under Chapter VIII establishing minimum KYC and sequence screening obligations across the Union. These acts should define scope (DNA and RNA synthesis, including benchtop and relevant in-house contexts), minimum outcome requirements, and reporting duties to national contact points.

Delegated acts should serve as the mechanism for updating Annex I, subject to procedural safeguards including consultation of the Advisory Group on Biosecurity, parliamentary and Council scrutiny, and proportionality review.

### 3.2 Embed standards and conformity mechanisms (short term)

The Commission should explicitly recognize international standards—particularly ISO 20688-2 and the work driven by the DNA Screening Standards Consortium (DSSC) in terms of its implementation, as well as established industry protocols such as those developed by the IGSC—as the primary reference framework for EU implementation. These standards should serve as the default benchmark for compliance across the Union. Recent inter-tool analysis of NIST screening datasets further highlights the importance of improving consistency and interoperability across nucleic acid sequence screening approaches (Laird, 2025).

A tiered conformity assessment framework should allocate responsibilities clearly across governance levels: economic operators would retain primary compliance responsibility; accredited third-party auditors would conduct proportionate, risk-based audits; national authorities would exercise supervisory and enforcement discretion; and the Commission would maintain an EU-wide registry of compliant providers and devices. Appeal mechanisms should be available for operators challenging audit findings or enforcement decisions.

### 3.3 Develop a centralised SOC service with oversight (short to medium term)

An EU-managed SOC service should support implementation once legal obligations are in place. It should include clear inclusion criteria, version control, secure access consistent with GDPR and NIS2, independent cybersecurity review, and a structured error-correction mechanism for false positives.

To reinforce legitimacy, oversight beyond the Advisory Group on Biosecurity should review governance procedures and proportionality impacts, for example, through periodic reporting or independent evaluation.

### 3.4 Operationalise risk-based decision frameworks (medium term)

A harmonised EU decision framework for SOC matches should be implemented through national supervisory structures. Providers retain primary responsibility for initial assessment, with Member States designating competent authorities for referrals and suspicious transaction reporting.

Providers should document decisions, customers should have access to review channels for denied orders, and safe harbor protections should shield good-faith reporting and denials from civil liability. Aggregated data should inform continuous refinement of screening parameters.

### 3.5 Integrate benchtop and in-house synthesis (medium term)

For new benchtop devices, embedded screening and authentication should be a condition for market entry. For legacy devices, transitional pathways should combine technical retrofits where feasible with institutional-level controls and time-limited exemptions under enhanced oversight.

Manufacturers should bear primary responsibility for device-level integration, with institutions responsible for operational controls in retrofit scenarios. Commission guidance should clarify liability allocation.

### 3.6 Align obligations with economic support (short term)

Compliance should be accompanied by proportionate financial instruments, including targeted grants and regulatory sandboxes linked to defined milestones. Incentives in EU research and strategic technology programmes should reward early compliance while avoiding structural distortions across Member States.

Where Member States establish regulatory sandboxes, these may be linked to defined innovation and biosecurity milestones and supported through EU financial instruments, provided that sandbox plans include risk mitigation, monitoring, and Commission notification mechanisms.

### 3.7 Consolidate legal and cybersecurity guidance (short term)

Integrated guidance should clarify the interaction between GDPR, NIS2, export controls, and Chapter VIII obligations. It should define data minimisation, retention, cybersecurity duties for SOC and screening providers, liability boundaries, and recommend Data Protection Impact Assessments for higher-risk actors.

### 3.8 Advance international interoperability (ongoing)

International engagement should follow establishment of the EU baseline, prioritising interoperability, mutual recognition pathways, and rights-respecting information sharing. Convergence should focus on outcome requirements rather than uniform institutional design.

International engagement should also take account of the WHO, 2025 Pandemic Agreement, Regional frameworks such as the ASEAN Leaders’ Declaration on Strengthening Regional Biosafety and Biosecurity (ASEAN, 2024) underscore the importance of interoperable international standards. International engagement should also take account of the WHO Pandemic Agreement, adopted by the 78th World Health Assembly in May 2025, which provides a multilateral framework for pandemic prevention, preparedness, and response and emphasises biosecurity, biosafety, and data protection standards as core commitments (World Health Organization, 2025). The agreement’s provisions on pathogen access and international information sharing are directly relevant to the EU’s efforts to promote responsible stewardship of synthetic nucleic acid sequences and to facilitate cross-border collaboration with third countries.

International engagement should also take account of the ongoing multilateral discussions on Digital Sequence Information (DSI) under the Convention on Biological Diversity (CBD) and its Nagoya Protocol. DSI — a placeholder term referring to data derived from de-materialised genetic resources, including nucleic acid sequence data — has been contested in international negotiations since 2016, when CBD Parties began considering whether such information should fall within the Nagoya Protocol’s Access and Benefit-Sharing (ABS) framework. The core tension arises from the fact that nucleic acid sequence data are freely accessible through major international online databases, which some biodiversity-rich nations have characterised as ‘digital biopiracy’, while the scientific community has argued that applying bilateral ABS permit requirements to DSI would be incompatible with modern research practices and would risk rendering sequence databases inoperable (Lawson et al., 2024; Muñoz–García et al., 2025). At COP15 in December 2022, CBD Parties agreed to establish a multilateral mechanism (MLM) for benefit-sharing from DSI, recognising open access as a key principle and thereby decoupling access from benefit-sharing (CBD Decision 15/9). The modalities were further defined at COP16 in Cali in November 2024 through Decision 16/2, which established the Cali Fund for the Fair and Equitable Sharing of Benefits from the Use of Digital Sequence Information on Genetic Resources, formally launched on 25 February 2025 (Convention on Biological Diversity, 2024; Muñoz–García et al., 2025).

These developments are directly relevant to the EU biosecurity screening framework proposed in this paper. The governance of pathogen-relevant sequence databases — heavily accessed by commercial synthesis providers, research laboratories, and biosecurity screening tools — sits at the intersection of biosecurity and biodiversity governance, creating a risk of incoherence between ABS obligations and biosecurity screening mandates if not carefully managed. The EU’s international engagement should therefore include coordination with the CBD process to ensure that benefit-sharing obligations and open-access data principles remain mutually supportive.

### 3.9 Establish monitoring and adaptive review (ongoing)

Monitoring should distinguish scientific threat assessment (Advisory Group mandate) from compliance effectiveness (Commission responsibility). The framework should include annual publication of aggregate compliance metrics, periodic independent evaluation of proportionality and innovation impacts, a formal review clause for delegated acts, and structured stakeholder feedback channels.

Monitoring should also take account of the Act’s recommendation to publish and update strategic project lists (art 62), annual reporting on regulatory sandboxes where established, and periodic review of delegated acts within the 5-year delegation cycle (Art 25a and 64).

For longer-term actions, monitoring and adaptive review should recognise good biosecurity practice and biosafety as core enablers of a safe and responsible innovation ecosystem, and hence a more secure and equitable EU bioeconomy. Embedding biosecurity and biosafety culture across the EU life sciences sector — through education, incentives, and institutional governance — is not merely a compliance objective but a foundational investment in the resilience and trustworthiness of the EU’s biotechnology industry. Long-term monitoring frameworks should therefore track not only compliance metrics but also indicators of biosecurity culture, capacity, and capability across the sector, consistent with the EU’s broader bioeconomy strategy.

## 4 Conclusion

The window for shaping global norms is narrow; early EU action on KYC and sequence screening will determine whether the Union leads or follows in governing the next-generation of biological technologies.

With targeted economic support, benchtop device integration requirements, legal guidance integrating the NIS2 Directive’s cybersecurity risk-management and reporting obligations, and coordination with the EU Dual-Use Regulation 2021/821, the EU can future-proof its bioeconomy, reinforce international norms, and demonstrate leadership in responsible innovation.

The Advisory Group on Biosecurity will play a crucial role in ensuring that the framework remains responsive to emerging biological threats, including those posed by AI models in biological applications.

Appropriate resourcing and expertise for the Advisory Group will be critical given the expected scale of implementation across EU Member States. The Group will be required to monitor biosecurity risks across 27 jurisdictions, assess rapidly evolving AI capabilities, and issue qualified alerts in a timely and scientifically credible manner. Sustainable funding, multidisciplinary expertise spanning biosecurity, AI, cybersecurity, and public health, and clear operational protocols will be essential to its effectiveness.

Through its mandate to monitor risks, assess AI model capabilities, and issue qualified alerts to the Commission regarding new biotechnology products of concern and AI models posing biological systemic risks, the Advisory Group provides an adaptive governance mechanism embedded within primary legislation.

Despite policy alignment, implementation presents significant risks. Capacity disparities across Member States may undermine consistent enforcement, particularly regarding technical screening capabilities, cybersecurity compliance and export classification expertise under the EU Dual-Use Regulation. Concrete capacity-building measures — including ECDC technical assistance, Commission-funded training for national competent authorities, and peer review mechanisms among Member States — will be essential to ensure consistent enforcement across the Union.

Smaller providers and benchtop manufacturers may face disproportionate compliance burdens without targeted financial and technical support.

Clear allocation of responsibilities among biosecurity, cybersecurity, export control, and AI authorities is essential to prevent duplication or enforcement gaps. Formal coordination and information-sharing mechanisms between the Advisory Group on Biosecurity and bodies established under the AI Act and Dual-Use Regulation will be critical.

Rapid technological evolution requires iterative updates to implementing and delegated acts, as well as adaptive technical guidance. Competitiveness concerns must also be addressed to prevent regulatory arbitrage. This requires international outreach, standards harmonisation and phased adoption pathways in partner regions.

By anchoring know-your-customer and sequence screening requirements within Chapter VIII of the EU Biotech Act, and operationalising them through a combination of delegated and implementing acts, the Union can translate high-level biosecurity principles into concrete, enforceable practice without undermining research and industrial competitiveness.

Embedding these measures within the broader architecture of the Act — including strategic project recognition, monitoring, the Biotechnology Investment Pilot, and structured use of regulatory sandboxes — ensures that biosecurity is not treated as a constraint on innovation but as an enabling condition for resilient biotechnology growth.

With the rapid adoption of emerging technologies and AI in the life sciences, there should also be a long-term view to incorporate screening practices and standard operating procedures (SOPs) into the Investigational Medicinal Product Dossier (IMPD), as referenced in Annex II, Section G of the relevant regulatory framework, for products that advance in clinical trials and potential licensure. Requiring evidence of compliant screening practices as part of the IMPD would serve as a powerful incentive for early adoption of best practices, embed biosecurity considerations into the product development lifecycle, and potentially provide a basis for preferential treatment in EU research and innovation funding programmes for products demonstrating compliance from the earliest stages of development.

Central to this approach is the integration of technical standards, an EU-managed sequences-of-concern service, and a risk-based conformity assessment model, supported by targeted economic measures and clear legal guidance, enabling consistent implementation across Member States.
